# Traitement chirurgical d'une luxation fracture parcellaire de la tete femorale pipkin II irreductible: à propos d'un cas

**DOI:** 10.11604/pamj.2014.18.253.2959

**Published:** 2014-07-26

**Authors:** Hatim Abid, Atif Mechchat, Mohammed El Idrissi, Mohammed Shimi, Abdelhalim El Ibrahimi, Abdelmajid El Mrini

**Affiliations:** 1 Service de chirurgie ostéo-articulaire B4, CHU Hassan II, Fès, Maroc

**Keywords:** Luxation traumatique, luxation irréductible, hanche, fracture, tête fémorale, ostéonécrose, arthrose, traumatic dislocation, irreducible dislocation, hip, fracture, femoral head, osteonecrosis, osteoarthritis

## Abstract

L'irréductibilité d'une luxation-fracture parcellaire de la tête fémorale est une entité pathologique rare dont le traitement est chirurgical. Dans la littérature, les avis des auteurs divergent concernant le choix de la voie d'abord, mais la conservation du fragment céphalique est la règle. Indépendamment de la qualité de la prise en charge, les résultats mauvais et passables sont très importants estimés à 50% au bout de 10 ans, et l’évolution arthrosique à long terme semble être inéluctable.

## Introduction

L'incidence des fractures parcellaires de la tête fémorale survenant lors des luxations de hanche est de 8 à 26%. Ces lésions sont rares et les variétés irréductibles le sont encore plus. Ces dernières compromettent gravement le pronostic fonctionnel futur de la hanche par un risque d'ostéonécrose et d'arthrose de 20% à 5 ans. Le traitement des luxations avec fractures parcellaires de la tète fémorale est difficile, autant pour le choix de la voie d'abord que pour l'attitude vis-à-vis du fragment fracturaire. Nous rapportons dans ce travail le cas d'une luxation-fracture de la tête fémorale Pipkin II irréductible.

## Patient et observation

**Figure 1:**
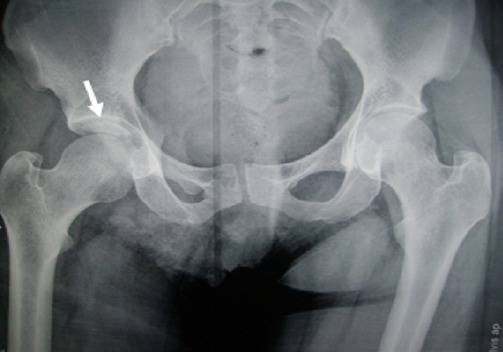
Radiographie du bassin de face objectivant une fracture pipkin II de la tête fémorale indentée sur le rebord postérieur du cotyle, le fragment céphalique est déplacé en polaire supérieur

**Figure 2:**
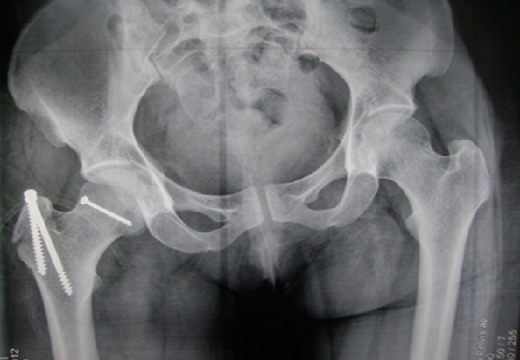
Radiographie du bassin de face en post opératoire immédiat, objectivant l'ostéosynthèse du fragment céphalique et de la trochantérotomie

Un patient de 37 ans, sans antécédents pathologiques notables, admis aux urgences pour traumatisme fermé de la hanche droite à deux heures d'un accident de la voie publique. L'examen clinique à l'admission retrouvait un patient conscient et stable sur les plans hémodynamique et respiratoire avec à l'examen locomoteur, une attitude vicieuse du membre inférieur droit en flexion de la hanche adduction rotation interne sans déficit vasculo- nerveux en distal. La radiographie standard a permis de poser le diagnostic d'une fracture luxation postérieure Pipkin II de la hanche droite, la tête étant indentée sur la paroi postérieure de l'acétabulum (Figure 1). Une tentative de réduction sous anesthésie générale était réalisée à 3 heures du traumatisme, sans succès. Dès lors, nous avons abordé l'articulation par voie postéro latérale: l'exploration avait objectivé une section partielle des muscles pélvi-trochantériens, une tète encochée sur le rebord postérieur du cotyle, avec un fragment céphalique expulsé en postérieur, solidaire au ligament rond. La tête fémorale était difficilement réintégrée à cause d'un effet boutonnière de la capsule, puis soigneusement repositionnée, après trochantérotomie, sur le fragment intra articulaire qui était antéro- inférieur et dont la fixation était obtenue par vissage en rappel au moyen d'une vis canulée 3.5 mm. Nous avons enchainé ensuite par l'ostéosynthèse de la trochantérotomie (Figure 2) et la réparation de la brèche capsulaire postérieure. Le patient a été mis sous traction trans tibiale pendant 3 semaines. L'appui s'est effectué à la fin de la sixième semaine. Au recul de 18 mois, le résultat fonctionnel est jugé excellent selon le score de Postel Merle d'Aubigné (PMA à 18 points), ceci sans anomalies radiologiques.

## Discussion

La luxation de la hanche irréductible demeure rare et ne représente que 3% des complications. Elle est due le plus souvent, comme dans notre cas, à une invagination capsulo labrale dans l'acétabulum [1, 2]. Cette irréductibilité fragilise le col fémoral, et augmente le risque de sa fracture lors des tentatives de réduction surtout en présence d'une fracture de la tête fémorale type II de Pipkin comme l'a rapporté Sy et al [3]. Dans ce cadre, le diagnostic de cette variété lésionnelle doit être fait initialement par le biais d'un bilan radiologique comportant des incidences du bassin de face, de profil, les ¾ alaire et obturateur, et s'associant idéalement à un scanner, ceci pour adopter des mesures préventives lors de la réduction par des manœuvres douces sur un malade bien curarisé.

La réduction orthopédique expose au risque de nombreuses complications, d'abord l'irréductibilité rapportée avec un pourcentage de 50% dans la série de Duquennoy et Butler [1], et de 57% dans celle de Vielpeau [4]. Ensuite la fracture du col qui est la complication la plus redoutable, celle-ci est souvent décrite dans les fractures de la tête fémorale Pipkin II, son incidence varie selon les auteurs de 12 à 27% [3, 4]. Puis, l'incarcération du fragment céphalique qui demeure rare, et qui est l'apanage des fractures céphaliques type I et II comminutives [3, 4]. Chez notre patient, la tentative de réduction s'est faite sous anesthésie générale par manœuvre de Bohler sans pour autant qu'elle réussisse.

Le traitement d'une luxation avec fracture parcellaire de la tête fémorale irréductible ne peut être que chirurgical. A ce propos, les avis des auteurs divergent sur le type de la voie d'abord à utiliser ainsi que sur l'attitude vis à vis du fragment céphalique.

En principe, l'abord chirurgical doit permettre un contrôle suffisant de la réduction de la luxation avec préservation maximale de la vascularisation de la tête fémorale [5, 6]. Dans ce cadre, Epstein Roeder et Delee utilisaient la voie postérieure qui assure un bon jour sur les variantes postérieures des luxations mais qui ne permet pas la fixation directe du fragment céphalique. Vielpeau quant à lui, préconisait la voie externe qui expose au risque de nécrose cutanée. Alors que Duquennoy utilisait en plus de la voie postérieure pour l'abord des luxations postérieures, la voie antérieure qui permet de contrôler la réduction puis la fixation du fragment de la tête détachée [1, 7, 8]. Chez notre malade, le choix de la voie postérieure associé à une trochantérotomie de digastrisation comme la faisait Duquennoy était justifié, d'une part puisqu'il s'agissait d'une luxation postérieure, et d'autre part pour préserver la vascularisation antérieure de la tête fémorale qui dépend de l'artère circonflexe antérieure. En plus, cette voie expose suffisamment l'articulation coxo-fémorale, facilite le contrôle digital du repositionnement du fragment céphalique tout en minimisant le risque de pseudarthrose du médaillon trochantérien.

Pour ce qui est du fragment céphalique, on note un nombre limité de publications traitant ce sujet. Pour Duquennoy, l'ablation du fragment fracturaire dans la variété Pipkin II est génératrice d'arthrose par une augmentation des pressions au niveau des zones d'appui articulaires, conséquence d'une diminution de la surface de contact entre la tête fémorale et la cavité cotyloïdienne [1]. De ce fait, l'attitude générale actuelle tend vers sa conservation et son ostéosynthèse afin d'espérer 60% de bons résultats à 10 ans [6–8]. Chez notre malade, nous avons adopté cette stratégie, et nous nous sommes aidés de la trochantérotomie pour d'abord replacer le fragment détaché en position antéro inférieur pour ensuite le fixer par vissage en rappel sous contrôle digitale.

En post opératoire, les différentes équipes préconisent une mise en traction continue pendant 3 semaines en moyenne avec une mise en décharge complète au cours de 2 mois. L'appui total n'est pas autorisé qu'après 3 mois.

**Figure 3:**
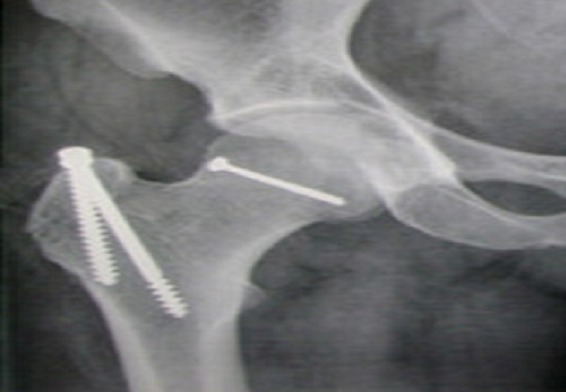
Radiographie de la hanche de face à 18 mois de recul sans signes d'ostéonécrose ni d'arthrose, la trochantérotomie étant consolidée

A long terme, les complications de la luxation fracture de la tête fémorale sont fréquentes avec 10% à 40% d'ostéonécrose dans les deux premières années, et 20% d'arthrose à 5 ans [9–11]. Jusqu'au dernier recul, notre patient ne présente pas de douleur à la mobilisation de la hanche opérée aussi bien en charge qu'en décharge, le suivi radiologique ne révèle pas d'anomalies (Figure 3).

## Conclusion

La variété Pipkin II des fractures luxations pure de la tête fémorale expose à un risque élevé de fracture du col fémoral lors de la tentative de réduction orthopédique de la luxation. Dans sa forme irréductible, le traitement est chirurgical. Ce dernier ne doit pas aggraver les lésions existantes. Pour cela, nous préférons La voie postéro latérale avec trochantérotomie assurant un jour suffisant de l'articulation, ainsi que la technique de vissage en rappel pour l'ostéosynthèse du fragment céphalique qui préserve la vascularisation du ligament rond.
